# Developing a novel immune infiltration-associated mitophagy prediction model for amyotrophic lateral sclerosis using bioinformatics strategies

**DOI:** 10.3389/fimmu.2024.1360527

**Published:** 2024-03-27

**Authors:** Rongrong Du, Peng Chen, Mao Li, Yahui Zhu, Zhengqing He, Xusheng Huang

**Affiliations:** ^1^ School of Medicine, Nankai University, Tianjin, China; ^2^ Department of Neurology, The First Medical Center, Chinese People's Liberation Army (PLA) General Hospital, Beijing, China; ^3^ Medical School of Chinese People's Liberation Army (PLA), Beijing, China; ^4^ Department of General Surgery & Institute of General Surgery, The First Medical Center of Chinese People's Liberation Army (PLA) General Hospital, Beijing, China; ^5^ Department of Neurology, Beijing Friendship Hospital, Capital Medical University, Beijing, China

**Keywords:** amyotrophic lateral sclerosis, mitophagy, immune infiltration, gene, prediction model, prognosis

## Abstract

**Background:**

Amyotrophic lateral sclerosis (ALS) is a fatal neurodegenerative disease, which leads to muscle weakness and eventual paralysis. Numerous studies have indicated that mitophagy and immune inflammation have a significant impact on the onset and advancement of ALS. Nevertheless, the possible diagnostic and prognostic significance of mitophagy-related genes associated with immune infiltration in ALS is uncertain. The purpose of this study is to create a predictive model for ALS using genes linked with mitophagy-associated immune infiltration.

**Methods:**

ALS gene expression profiles were downloaded from the Gene Expression Omnibus (GEO) database. Univariate Cox analysis and machine learning methods were applied to analyze mitophagy-associated genes and develop a prognostic risk score model. Subsequently, functional and immune infiltration analyses were conducted to study the biological attributes and immune cell enrichment in individuals with ALS. Additionally, validation of identified feature genes in the prediction model was performed using ALS mouse models and ALS patients.

**Results:**

In this study, a comprehensive analysis revealed the identification of 22 mitophagy-related differential expression genes and 40 prognostic genes. Additionally, an 18-gene prognostic signature was identified with machine learning, which was utilized to construct a prognostic risk score model. Functional enrichment analysis demonstrated the enrichment of various pathways, including oxidative phosphorylation, unfolded proteins, KRAS, and mTOR signaling pathways, as well as other immune-related pathways. The analysis of immune infiltration revealed notable distinctions in certain congenital immune cells and adaptive immune cells between the low-risk and high-risk groups, particularly concerning the T lymphocyte subgroup. ALS mouse models and ALS clinical samples demonstrated consistent expression levels of four mitophagy-related immune infiltration genes (*BCKDHA*, *JTB*, *KYNU*, and *GTF2H5*) with the results of bioinformatics analysis.

**Conclusion:**

This study has successfully devised and verified a pioneering prognostic predictive risk score for ALS, utilizing eighteen mitophagy-related genes. Furthermore, the findings indicate that four of these genes exhibit promising roles in the context of ALS prognostic.

## 1 Introduction

Amyotrophic lateral sclerosis (ALS) is a neurodegenerative disease affecting upper and lower motor neurons, characterized by progressive muscle weakness, atrophy leading to paralysis, and eventual fatality. ALS has insidious onset, rapid progression, heterogeneous clinical manifestations, and currently lacks effective treatment options. The majority of patients succumb to respiratory failure within 3-5 years of onset (1, 2), imparting a heavy burden on patients, their families, and society. Presently, ALS diagnosis primarily relies on neurophysiological and neuroimaging examinations, yet early diagnosis and treatment remain challenging. Thus, the identification of practical early diagnostic markers and the construction of a more accurate ALS diagnostic and prognostic model may offer new hope for ALS patient treatment.

Mitophagy is a crucial process for mitochondrial quality control, its malfunction leads to the accumulation of defective mitochondria, posing a risk of damage to high-energy-demanding neuronal cells. Studies have indicated that mitophagy dysfunction is a key factor in the occurrence and progression of various neurodegenerative diseases such as ALS, Parkinson’s disease, and Alzheimer’s disease (3). Furthermore, research has pointed out that energy metabolism disturbances resulting from mitochondrial dysfunction are central to the pathophysiology of ALS (4, 5). To date, nearly 40 ALS-related genes have been identified. Some of these genes (*OPTN, SQSTM1/p62TBK1, SOD1, C9ORF72, VCP*) are directly or indirectly associated with the mitophagy pathway, influencing different stages of the mitophagy process (6, 7). The findings of these studies indicate that mitophagy is a significant factor in the pathogenesis of ALS. Nevertheless, the specific involvement of mitophagy-related genes (MRGs) in the progression of ALS remains largely unexplored. Consequently, a comprehensive investigation into mitophagy-related markers in ALS using bioinformatics tools may facilitate the discovery of novel biomarkers with therapeutic potential for ALS. Furthermore, it has been observed that mitophagy also contributes to the regulation of the immune response. Mitophagy has the potential to exert an anti-inflammatory effect by suppressing the excessive production of interleukin (IL)-1β and IL-18 (8). Dysregulation of mitophagy, on the other hand, triggers inflammation by activating the pyrin domain-containing protein 3 (NLRP3) inflammasomes, resulting in an overexpression of IL-1β and IL-18 (9, 10). Additionally, the release of mitochondrial DNA has been shown to promote the transcription of various inflammatory cytokines, including tumor necrosis factor (TNF-α) and IL-6 (11). The unique anatomy of spinal motor neurons makes ALS particularly susceptible to peripheral immune responses (12). Blood monocytes and macrophages react to degenerating motor nerves, producing cytokines that can act locally or travel through the blood-brain barrier to the central nervous system. These cytokines are being studied as potential early biomarkers for ALS (13). A meta-analysis on ALS revealed a consistent trend towards elevated blood levels of pro-inflammatory cytokines, including IL-1B, IL-6, and TNF, which are known to be produced by reactive monocytes/macrophages (13–15).

The objective of this study is to conduct multifaceted analyses of different datasets related to ALS in the GEO database. Limma and Spearman correlation analyses were used to identify mitophagy-related DEGs in ALS and filter for mitophagy genes. Subsequently, machine learning methods (forest plot, univariate analysis, and least absolute shrinkage and selection operator (LASSO) regression) were used to filter and identify prognostic markers and construct a risk model. Finally, we used Gene Set Enrichment Analysis (GSEA) and Receiver Operating Characteristic (ROC) curve analysis to create and evaluate the prediction model molecule drugs. We also collected ALS mouse models and ALS patients to confirm the expression levels of the model’s feature genes. The study aimed to elucidate the relationship between mitophagy, ALS, and immune infiltration by constructing a mitophagy-associated prediction model and examining its association with immune infiltration. Our study sheds new light on the role of mitophagy and immune inflammation in predicting the prognosis and diagnosis of ALS.

## 2 Materials and methods

### 2.1 Acquisition and preprocessing of expression profiling data

The ALS patient dataset was sourced from the Gene Expression Omnibus (https://www.ncbi.nlm.nih.gov/geo/), with the candidate dataset being selected based on specific inclusion criteria, including ALS diagnosis, human gene expression profile, availability of follow-up information (survival information), and related clinical data. The gene expression data from GSE112676 and GSE112680, obtained from Illumina HumanHT-12 V3.0 and HumanHT-12 V4.0 expression bead chip arrays, were incorporated into the study. The dataset GSE112676 consisted of 233 ALS samples and 508 control (CON) samples, while the dataset GSE112680 cohort, comprised 164 ALS samples and 137 control samples. Survival information was available for all 397 ALS patients. Demographic details of the cohorts have been previously documented (16). In summary, the ALS and CON groups exhibited a higher proportion of male participants (≥58.54%) with mean ages of 63.92 and 63.58, respectively. The majority of patients (>60%) presented with spinal-onset ALS rather than bulbar-onset ALS. The GSE112680 cohort had a higher percentage of individuals with C9orf72 repeat expansions (12.8% vs. 5.2%). Survival was operationally defined as the duration from disease onset to death, tracheostomy, or noninvasive ventilation (16). According to this operationalization, the median survival time was 2.42 years, with 50% of patients surviving. As shown in **Table 1**.

**Table 1:** Baseline characteristics.

Variable	Overall, N = 397^1^	Cohort		p-value^2^
		Training cohort N = 233 (59%)^1^	Validation cohort N = 164 (41%)^1^	
**Age of onset**	63.78 [55.60,70.72]	63.92 [56.37,70.75]	63.58 [54.81,70.66]	0.696
**Survival time (years)**	2.42 [1.59, 3.52]	2.50 [1.64, 3.79]	2.34 [1.56, 3.35]	0.263
Sex				0.642
Female	158 (39.80%)	90 (38.63%)	68 (41.46%)	
Male	239 (60.20%)	143 (61.37%)	96 (58.54%)	
Site of onset				0.420
Bulbar	146 (36.78%)	90 (38.63%)	56 (34.15%)	
Spinal	251 (63.22%)	143 (61.37%)	108 (65.85%)	
Status				0.022
Dead	342 (86.15%)	209 (89.70%)	133 (81.10%)	
Survival	55 (13.85%)	24 (10.30%)	31 (18.90%)	

^1^Median [IQR]; n (%).

^2^Wilcoxon rank sum test; Pearson’s Chi-squared test.

The methodology employed by Swindell et al. (16) was consulted for a comprehensive account of the data processing procedures and outcomes, with particular attention to mitigating platform-specific biases and batch confounders. The relevant GSE dataset was obtained by directly downloading the preprocessed and standardized probe expression matrix. Gene probes were converted to gene symbols utilizing the respective annotation profiles within each dataset. Normalization of gene expression values and the generation of normally distributed expression values were achieved using the ‘limma’ package in R software. In cases where multiple probes corresponded to the same gene, the final gene expression value was determined by calculating the average expression value.

### 2.2 Differential expression pattern analysis

The differential expression analysis between the ALS and CON groups was conducted utilizing the GSE112676 dataset. This analysis was performed employing the R package limma (version V-3.84.3, https://www.bioconductor.org/packages/release/bioc/html/limma.html) within R version 4.3.0. Gene-specific information, including P-values and logFC values, was obtained and analyzed using the Benjamini & Hochberg method to account for multiple tests. This method yielded adjusted p-values (adj.P.Value). Differentially expressed genes (DEGs) were identified using significant differences in fold change and statistical significance, with a threshold set at adj.P.Val < 0.05.

### 2.3 Mitophagy-related gene screening

To investigate the correlation between mitophagy genes and DEGs, a collection of thirty-four mitophagy genes associated with ALS was identified based on the Relevance score > 1.5 in the Genecards database (http://www.genecards.org/), searched using the keyword “Mitophagy”. Further analysis was performed to investigate the relationship between these mitophagy genes and the identified DEGs. the corrplot package (v-0.90, https://cran.r-project.org/web/packages/corrplot/vignettes/corrplot-intro.html) was utilized. Genes that exhibited a significant correlation (P<0.05) and a correlation coefficient (r) exceeding 0.3 were deemed relevant and selected as relevant genes. These relevant genes, in conjunction with the mitophagy genes, were designated as mitophagy-related genes for subsequent analysis.

### 2.4 Identification of prognostic significance genes

Utilizing the aforementioned mitophagy-related genes, conducted univariate Cox regression analysis using the survival-V3.2.13 package (https://github.com/therneau/survival) to identify potential genes associated with ALS. The corresponding P-value for each gene was assessed, and a threshold of P<0.05 was established for evaluation purposes.

### 2.5 Development and validation of risk scoring

The glmnet package (V-4.1-2, https://cran.r-project.org/web/packages/glmnet/index.html) was utilized to perform LASSO Cox analysis on the candidate prognostic genes obtained from the training set GSE112676. This analysis aimed to select feature genes with nonzero regression coefficients to construct a risk-scoring model. To assess the accuracy of the risk scoring model, the prognostic model construction method was followed, wherein the Risk score for each diseased sample was calculated by adding the expression level of each gene multiplied by its corresponding coefficient (e.g., Risk score = (expression level of gene A * coefficient of gene A) + (expression level of gene B * coefficient of gene B) +…), the Risk score values were calculated for each diseased sample in the training dataset GSE112676. Subsequently, employing the optimal threshold value derived from the median Risk score, the diseased samples within the training dataset GSE112676 were partitioned into two groups: High_Risk (comprising samples with a Risk score greater than or equal to the median Risk score) and Low_Risk (comprising samples with a Risk score lower than the median Risk score). The survival package in R4.1.0 was utilized to generate survival prognostic curves, enabling the evaluation of the relationship between the aforementioned grouping of High_Risk and Low_Risk samples and the actual survival prognosis information. The log-rank test was used to determine the statistical significance of the survival prognosis disparity among the two groups. Furthermore, the prognostic significance of the feature genes in the training dataset GSE112676 was assessed by calculating the Area Under Curve (AUC) values of the ROC curve at 5, 7, and 10 years. It should be noted that the AUC for validation sets at 1, 2, and 3 years is 0, as the classification data for survival time in these sets consists solely of either all 1 or all 0. Similarly, the accuracy of the risk scoring model was confirmed in the external validation dataset GSE112680 from GEO using the appropriate signature construction method.

### 2.6 The relationship between high- and low-risk groups and immune infiltration

Mitophagy and immunity are closely related, and peripheral immune cells play a significant role in disease progression in ALS patients (17). Therefore, it is meaningful to link with immune infiltration in the study. CIBERSORT analysis (accessible at https://cibersortx.stanford.edu/) was used to estimate the proportions of 22 human immune cell subsets based on gene expression data. Finally, the disparities in the distribution of TMB scores and proportions of immune cell infiltration between the high-risk and low-risk groups were evaluated using the Wilcoxon test.

### 2.7 Prognostic model genes and immune correlation analysis

Conducting correlation analysis on the feature genes and immune infiltration proportions was performed using the corrplot package (v-0.90, https://cran.r-project.org/web/packages/corrplot/vignettes/corrplot-intro.html).

### 2.8 Differences in checkpoint genes and HLA family genes between high-risk and low-risk groups

The expression data of common immune checkpoint genes and the Human Leukocyte Antigen (HLA) gene family, which comprises 17 HLA genes, were extracted from the training set GSE112676. The differential expression of immune checkpoint genes and the HLA gene family between the high-risk and low-risk groups was compared using the Wilcoxon test.

### 2.9 Analysis of molecular mechanisms between high- and low-risk groups

To conduct a more comprehensive examination of the molecular mechanisms underlying the distinction between high- and low-risk groups in ALS, 50 hallmark gene sets from the MSigDB database (http://www.gsea-msigdb.org/gsea/index.jsp) were obtained. The hallmark enrichment scores were computed using the GSEA function from the clusterProfiler package (V-4.6.2, https://www.bioconductor.org/packages/release/bioc/html/clusterProfiler.html).

### 2.10 Differential gene selection between high- and low-risk groups

Differential expression analysis was conducted on the high- and low-risk groups using the GSE112676 dataset. The limma package (V-3.84.3, available at https://www.bioconductor.org/packages/release/bioc/html/limma.html) in R version 4.3.0 was employed to obtain gene-specific information, including P-values and logFC values. Furthermore, multiple testing correction was performed using the Benjamini & Hochberg method to derive adjusted p-values (adj.P.Value). DEGs were identified using the following criteria: adj.P.Value < 0.05 and |logFC| > 1.5.

### 2.11 Enrichment analysis of differential genes between high- and low-risk groups

The DEGs obtained from the high- and low-risk groups were subjected to Gene Ontology (GO) analysis using the clusterProfiler package (V-4.6.2, https://www.bioconductor.org/packages/release/bioc/html/clusterProfiler.html). This analysis encompassed the categories of cellular component (CC), molecular function (MF), and biological process (BP). Additionally, the Kyoto Encyclopedia of Genes and Genomes (KEGG) pathway enrichment analysis was conducted. A significance threshold of P-value < 0.05 was applied to ascertain significant enrichment.

### 2.12 Diagnostic analysis of model genes

The differential expression of the feature genes between the ALS and CON groups was assessed through the utilization of the Wilcox test on both the training dataset GSE112676 and the validation dataset GSE112680. The pROC package (V-1.18.2, https://www.rdocumentation.org/packages/pROC/versions/1.18.2) was employed to generate ROC curves for the feature genes.

### 2.13 Real-time polymerase chain reaction

A group of 10 patients diagnosed with ALS and 10 healthy individuals of the same age and gender were selected for the study. Blood samples were collected from both groups to investigate the gene expression patterns of specific genes in a diagnostic model using real-time quantitative polymerase chain reaction (RT-qPCR). The ALS mouse model (B6SJL-Tg(SOD1^G93A^)) was acquired from The Jackson Laboratory in the United States, and RT-qPCR was conducted on the lumbar spinal cord of SOD1^G93A^ mice to evaluate the gene expression trends of characteristic genes in the disease model. The procedure for collecting the samples was approved by the Ethics Committee of the First Medical Center of Chinese PLA General Hospital. Total RNA was isolated from peripheral blood and spinal cord tissues using the RNAprep Pure High-Efficiency Total RNA Extraction Kit and the RNAeasy Fast Animal Tissue/Cell Total RNA Extraction Kit, respectively. RT-PCR was conducted using the FastKing One-Step RT-PCR Kit. Gene primers were designed using Primer 5 and synthesized by Biomed. GAPDH expression was utilized as an internal control, and relative expression was determined using the 2^–ΔΔCt^ method.

### 2.14 Statistical analysis

All statistical analyses were conducted using R software (version 4.1.0 and 4.3.0) and R studio (Version 3.84.3). The Wilcoxon test was utilized to compare the proportions of immune cell infiltration between the high- and low-risk groups and to analyze the differential expression of the feature genes in the ALS and CON groups. LASSO-Cox regression was used for feature gene selection. The log-rank test was then conducted to compare the survival rates of low- and high-risk groups. Univariate Cox regression analyses were also performed to identify genes that may be associated with ALS. A two-tailed P value<0.05 was considered statistically significant, with some exceptions where a specific P value was set.

## 3 Results

### 3.1 Acquisition and preprocessing of expression profile data

**Figure 1:**
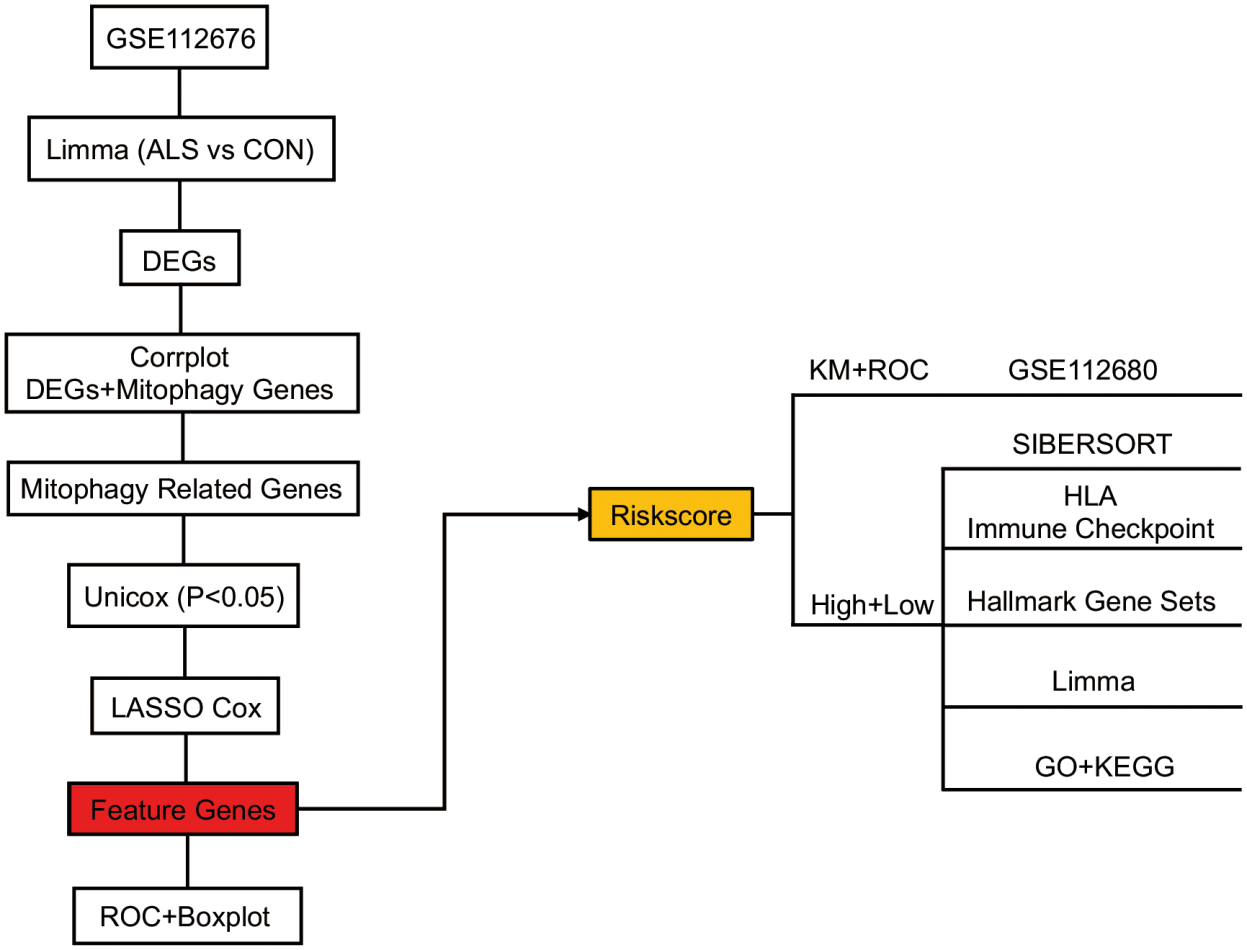
Overview of the schematic diagram of this study.

The study’s schematic diagram is presented in **Figure 1**. The training set, GSE112676, was acquired and comprised 508 control samples and 233 ALS samples, all of which had survival information available. The validation set, GSE112680, consisted of 137 control samples and 164 ALS samples, all of which had survival information available. To explore the association between mitophagy genes and differentially expressed genes (DEGs), a set of thirty-four mitophagy genes (**Supplementary Materials 1**) linked to ALS was identified using a Relevance score > 1.5 in the Genecards database (http://www.genecards.org/), utilizing the keyword “Mitophagy”. Subsequent analysis was conducted to examine the connection between these mitophagy genes and the identified DEGs, employing the corrplot package (v-0.90, https://cran.r-project.org/web/packages/corrplot/vignettes/corrplot-intro.html). Genes demonstrating a significant correlation (P<0.05) and a correlation coefficient (r) greater than 0.3 were considered pertinent and chosen as relevant genes. These relevant genes, in conjunction with the mitophagy genes, were designated as mitophagy-related genes for subsequent analysis.

### 3.2 Identification of prognostic significance genes in mitophagy-related genes

**Figure 2:**
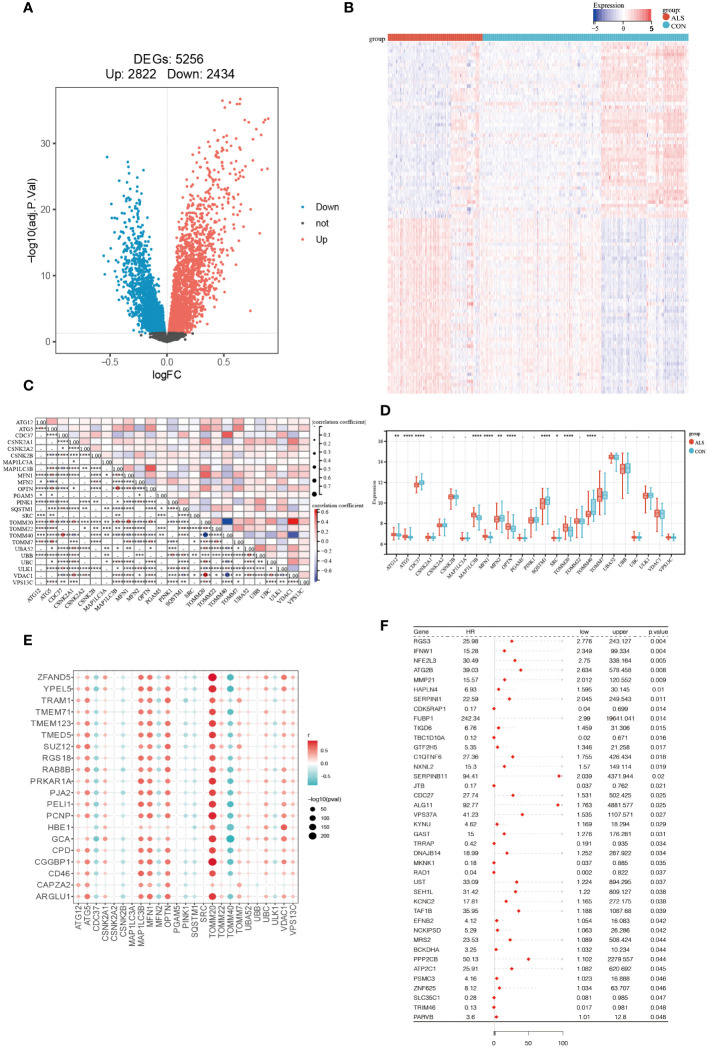
Identification of Prognostic Significance Mitophagy-Related Genes in ALS. **(A)** Differential analysis volcano plot (red represents significantly upregulated genes, blue represents significantly downregulated genes, and black represents non-significant genes). **(B)** Heatmap of differentially expressed mitophagy-related genes between ALS and control samples. **(C)** The correlation of 25 mitophagy-related genes. **(D)** The expression levels of 25 mitophagy genes in the CON group vs. the ALS group. **(E)** Scatter plot showing the correlation between the top 20 DEG genes with the largest logFC and mitophagy genes. The redder the point, the stronger the positive correlation; the bluer the point, the stronger the negative correlation. The larger the shape of the point, the smaller the p-value; the smaller the shape, the larger the p-value. **(F)** Forest plot displaying the results of the univariate Cox analysis. ****P<0.0001, ***P<0.001, **P<0.01, *P<0.05, ns P>0.05.

Firstly, the limma package was used to analyze the DEGs between CON and ALS on all expression matrices of the training set GSE112676, with adjust P-value <0.05 as a significance threshold. A total of, 5256 DEGs were identified from this analysis, including, 2822 upregulated genes and, 2434 downregulated genes (**Figure 2A**). Similarly, we analyzed differentially expressed genes in the validation set. A total of 1079 genes were down-regulated and 1300 genes were up-regulated (**Supplementary Figure 1**). Additionally, a heatmap was employed to visually represent the DEGs (**Figure 2B**). According to the mitophagy database, a total of 34 genes involved in the process of mitophagy were obtained (**Supplementary Material 1**). After comparing training set GSE112676 genes with all mitophagy-related genes, 25 overlapping genes were identified (**Supplementary Material 2**). A Spearman correlation analysis was performed on a set of 25 genes associated with mitophagy. The correlation among these 25 mitophagy-related genes is depicted in **Figure 2C**, while an examination of the expression levels of these 25 mitophagy genes in the CON group compared to the ALS group is presented in **Figure 2D**. Among them, the expression level of eight genes (*ATG12, ATG5, MAP1LC3B, MFN1, OPTN, SRC, TOMM20, TOMM7*) was upregulated, while the expression level of four genes (*CDC37, MFN2, SQSTM1, TOMM40*) were downregulated. Using the corrplot package, Spearman correlation analysis was conducted between the, 5256 DEGs and the 25 mitophagy genes. In light of the extensive number of genes, a correlation heatmap analysis specifically targeted the top 20 DEGs with the highest logFC values, as well as the genes associated with mitophagy (**Figure 2E**). Subsequently, employing a significance threshold of P < 0.05 and |r| > 0.3, 4405 genes were identified, comprising, 4383 DEGs and 22 mitophagy genes (*MFN2, OPTN, MAP1LC3B, ATG12, PINK1, MFN1, TOMM20, TOMM7, TOMM40, ULK1, ATF5, CDC37, CSNK2A1, UBC, VDAC1, UBA52, SQSTM1, TOMM22, CSNK2B, VPS13C, UBB, CSNK2A2*) (**Supplementary Material 3**). To further investigate their prognostic potential, univariate Cox analysis on the aforementioned, 4405 genes, led to the discovery of 40 genes with significant prognostic value, as indicated by a P-value < 0.05 (**Figure 2F**).

### 3.3 Construction and validation of risk score

**Figure 3:**
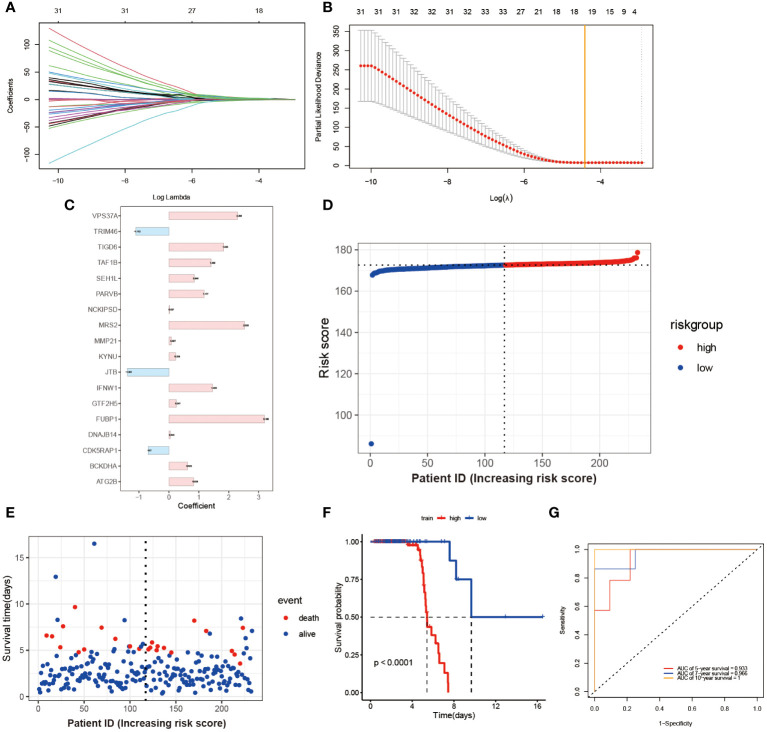
Construction and Validation of Risk Score Model. **(A)** Each curve in the figure represents the trajectory of each independent variable coefficient. The y-axis represents the coefficient values, while the lower x-axis represents log(λ) and the upper x-axis represents the number of non-zero coefficients in the model at that time. **(B)** The lowest point on the curve indicates the optimal lambda, which is the intersection between the yellow line and the red dot. **(C)** The histogram shows the distribution of coefficient values for the selected features. The distribution of risk scores **(D)** and survival times for each patient sample **(E)** in the training set GSE112676 is shown. The Kaplan-Meier survival curves **(F)** and time-dependent ROC curves **(G)** are presented for both high and low-risk groups.

Through the utilization of LASSO-Cox regression analysis on the aforementioned candidate genes, a subset of 18 genes (*VPS3TA, TR1M46, TIGD6, TAF1B, SEH1L, PARVB, NCK1PSD, MRS2, MMP21, KYNU, JTB, IFNW1, GTF2H5, FUBP1, DNAJB14, CDK5RAP1, BCKDHA, ATG2B*) was identified for the development of a prognostic risk score based on the minimal criteria of λ (**Figures 3A, B**). The risk score for each sample was calculated using the formula: Risk score = [(2.298 x *VPS3TA* expression value) + (-1.112 x *TR1M46* expression value) + (1.835 x *TIGD6* expression value) + (1.409 x *TAF1B* expression value) + (0.846 x *SEH1L* expression value) + (1.177 x *PARVB* expression value) + (0.027 x *NCK1PSD* expression value) + (2.525 x *MRS2* expression value) + (0.087 x *MMP21* expression value) + (0.228 x *KYNU* expression value) + (-1.391 x *JTB* expression value) +(1.456 x *IFNW1* expression value) + (0.247 x *GTF2H5* expression value) + (3.198 x *FUBP1* expression value) + (0.043 x *DNAJB14* expression value) + (-0.7 x *CDK5RAPI* expression value) + (0.623 x *BCKDHA* expression value) + (0.828 x *ATG2B* expression value)] (**Figure 3C**). Subsequently, the 164 patients were stratified into two risk groups based on the median risk score, with 113 patients categorized as low-risk and 51 patients as high-risk (**Figure 3D**). Patients in the high-risk group exhibited higher mortality rates (**Figure 3E**), as evidenced by the Kaplan-Meier curve demonstrating superior survival rates among patients in the low-risk group (P < 0.0001, **Figure 3F**). Time-dependent ROC analysis of the risk score revealed that the area under the curve (AUC) was 0.933, 0.966, and 1 for 5-, 7-, and 10-year survival, respectively (**Figure 3G**).

**Figure 4:**
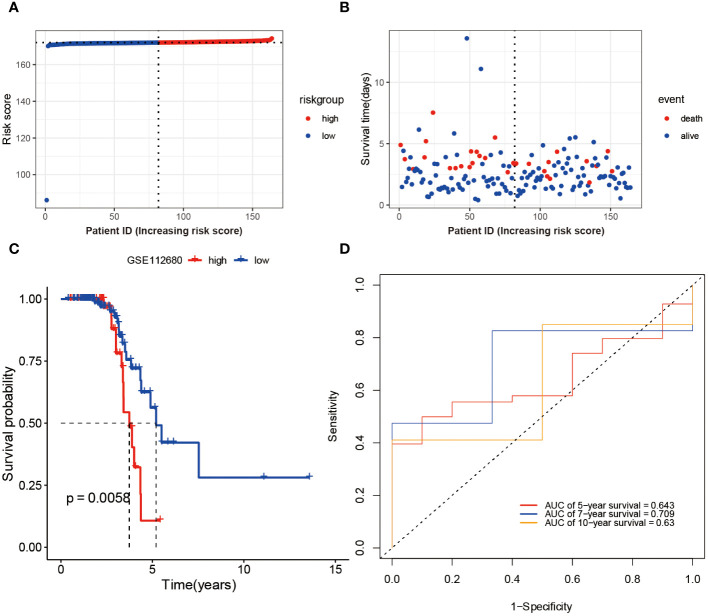
Validation of Risk Score Model in GSE112680 geneset. Risk scores **(A)** and survival time distribution **(B)** for each patient sample in the validation set GSE112680 were plotted. Kaplan-Meier survival curves **(C)** for the high and low-risk groups, as well as time-dependent ROC curves **(D)**, were generated.

To Validate the prognostic risk score, patients in the validation set, GSE112680, were divided into two groups based on the median risk score (**Figure 4A**). Patients in the high-risk groups experienced a higher incidence of mortality (**Figure 4B**), as evidenced by the Kaplan-Meier curve demonstrating significantly greater survival rates among patients in the low-risk group compared to those in the high-risk group (validation set: P = 0.0058 **Figure 4C**). These findings demonstrate the reliability of the ROC analysis and indicate that the risk scoring model is highly feasible, with AUC values of 0.643, 0.709, and 0.63 for 5-year, 7-year, and 10-year predictions, respectively (**Figure 4D**).

### 3.4 Relationship between high- and low-risk groups and immune response

**Figure 5:**
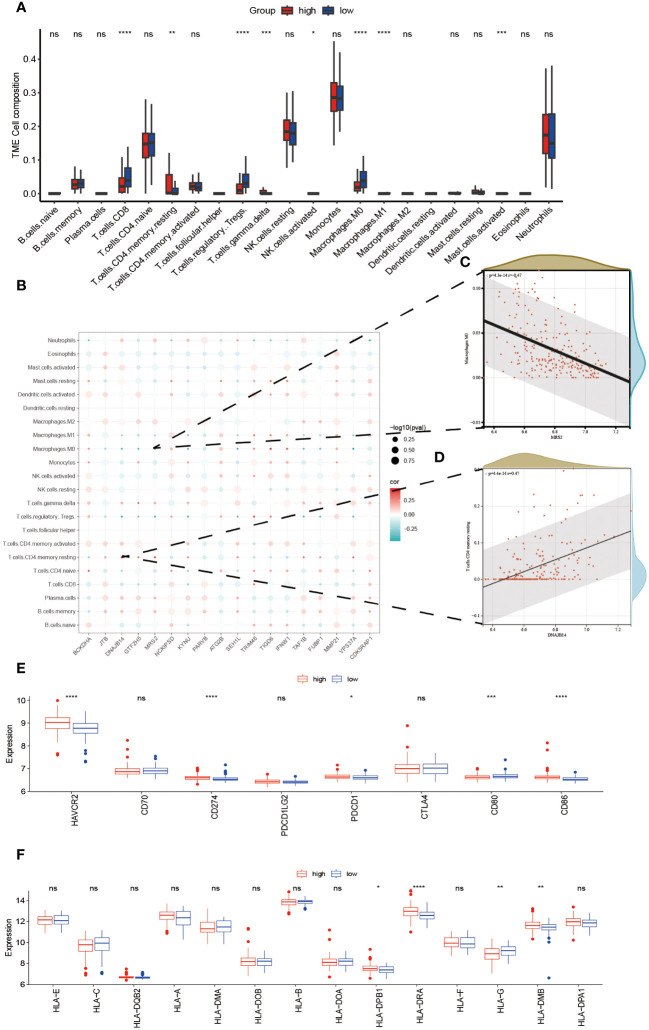
Relationship Between High- and Low-Risk Groups and Immune Response. The infiltration abundance distribution using the CIBERSORT algorithm **(A)**. **(B)** Scatter plot illustrating the correlation between the expression levels of model genes and the levels of immune cell infiltration. The color of the points indicates the strength of positive correlation (red) or negative correlation (blue). The size of the points reflects the p-value, with larger points indicating smaller p-values. **(C)** MRS2 showed a marked negative correlation with M0 macrophages. **(D)** DNAJB14 exhibited a significant positive correlation with T cells CD4 memory. Box plots displaying the expression levels of immune checkpoint genes **(E)** and HLA genes **(F)** between high- and low-risk groups. ****P<0.0001, ***P<0.001, **P<0.01, *P<0.05, ns P>0.05.

An investigation was conducted to determine the connection between immune-related genes and ALS High- and Low-Risk groups through the analysis of immune cell infiltration. The CIBERSORT algorithm was used to estimate the relative infiltration abundance of immune cell types in each sample (**Figure 5A**). Additionally, the differences in immune cells between the two risk groups were compared and their significance was assessed using the Wilcoxon test. A total of 22 immune cells infiltrating between the ALS high- and low-risk groups were screened with values of p<0.05, as shown in (**Figure 5A**). Patients with higher scores exhibited significantly elevated levels of CD4 memory resting Tcells, gamma delta Tcells, and M1 Macrophages while showing relatively lower proportions of CD8 Tcells, M0 macrophages, and NK cells, Mast cell activated and regulatory Tregs T cells. Additional examination of the 18 mitophagy-related genes that were chosen, along with their association with immune cells (**Figure 5B**), unveiled a noteworthy inverse association between MRS2 and M0 macrophages (r=-0.47, P<0.001, **Figure 5C**). Conversely, there was a noteworthy positive correlation between DNAJB14 and resting memory CD4 T cells(r=0.47, P<0.001, **Figure 5D**). Further analysis of eight immune checkpoint genes showed that the expressions of *HAVCR2, CD274, PDCD1*, and CD86 were elevated and the expressions of CD80 were relatively lower in the high-risk group of ALS (**Figure 5E**). Since HLA family genes play a crucial role in immune response, we also analyzed the association between HLA family genes and high- and low-risk groups. we found that patients with higher scores exhibited significantly elevated levels of *HLA-DPB1, HLA-DRA*, and *HLA-DMB* while showing relatively lower proportions of *HLA-G* (**Figure 5F**).

### 3.5 Molecular mechanism analysis between high- and low-risk groups

**Figure 6:**
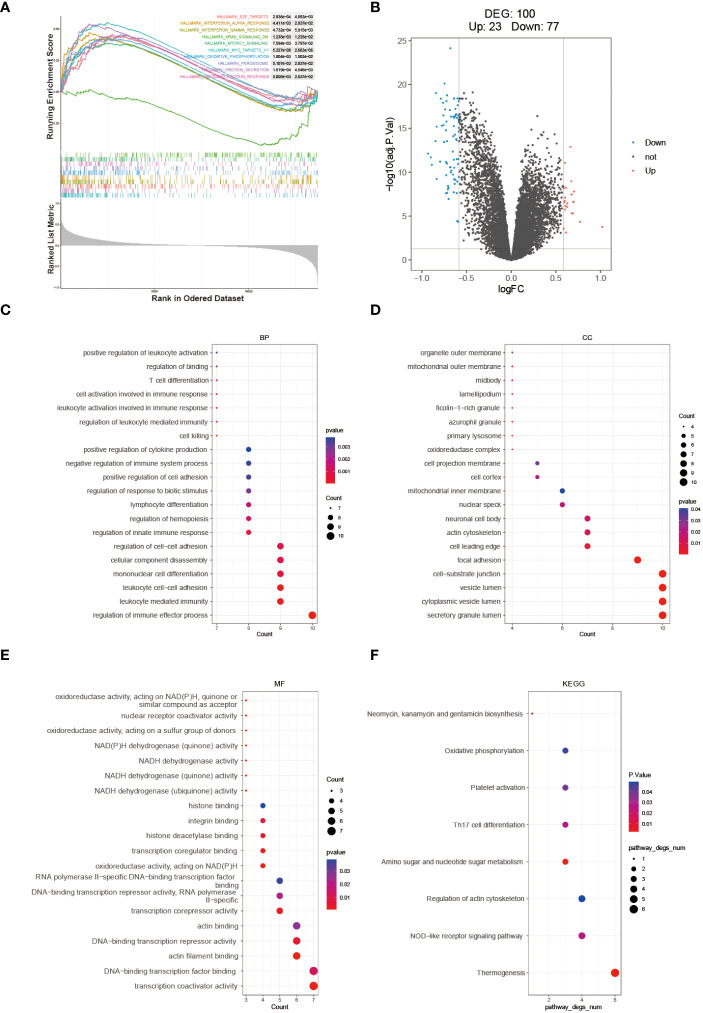
Molecular mechanism analysis between high- and low-risk groups. **(A)** GSEA Enrichment Analysis Plot. **(B)** Volcano plot depicting the differential expression between high- and low-risk groups. GO **(C–E)** and KEGG **(F)** enrichment analysis results are displayed, with stronger significance indicated by redder color and lower significance indicated by bluer color.

The enrichment scores for multiple hallmark pathways were calculated using GSEA. Among these pathways, a total of 10 showed significant enrichment, with normalized enrichment scores (NES) greater than 0 indicating activation in the high-risk group and NES less than 0 indicating inhibition in the high-risk group (**Figure 6A**). To further analyze the high- and low-risk groups in the training set (GSE112676), differential analysis was performed using the limma package with the criteria of adj.P.Val < 0.05 and |logFC| > 1.5. This analysis identified 100 DEGs, including 23 upregulated and 77 downregulated genes (**Figure 6B**). GO and KEGG pathway enrichment analyses were performed on these 100 DEGs using R software to explore their potential biological functions and pathways. The results of GO functional analysis revealed that the most significant items of GO enrichment included leukocyte mediated immunity, leukocyte cell-cell adhesion, regulation of immune effector process in biological process (BP), secretory granule lumen, cytoplasmic vesicle lumen, mitochondrial outer membrane in cellular component (CC) and transcription coactivator activity in molecular function (MF) (**Figures 6C–E**). The results of KEGG pathway enrichment analysis showed that they were mainly enriched in Thermogenesis, NOD-like receptor signaling pathway, and Amino sugar and nucleotide sugar metabolism (**Figure 6F**).

### 3.6 Diagnostic analysis of model genes and validation of the four key DEGs in SOD1^G93A^ mice lumbar spinal cord tissue and clinical samples

**Figure 7:**
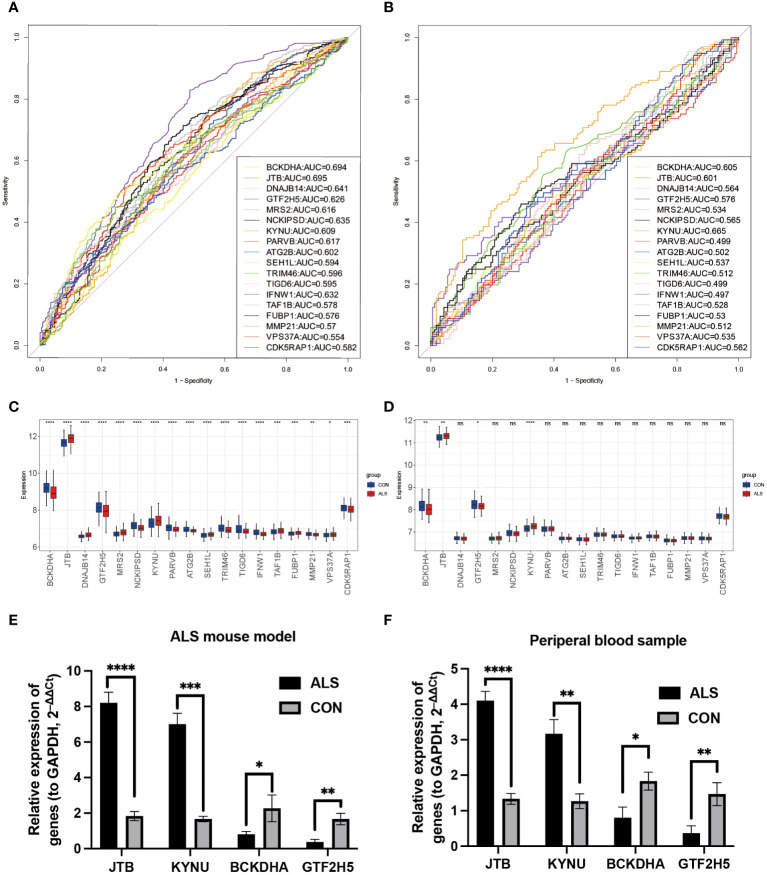
Diagnostic analysis of model genes and Validation of the four key DEGs in the lumbar spinal cord tissue of SOD1^G93A^ mice and clinical samples. In the training set GSE112676, the ROC plot to assess disease prediction performance **(A)**, as well as box plots were used to depict the differential distribution of model genes **(C)**. **(B, D)** The same analysis was performed in the validation set GSE112680. Additionally, the expression levels of four key genes were validated by RT-qPCR in ALS mice lumbar spinal cord **(E)** and peripheral blood samples from ALS patients **(F)**. ****P<0.0001, ***P<0.001, **P<0.01, *P<0.05, ns P>0.05.

To verify whether the model genes we screened have significant differences in diagnosis, ROC curves were drawn based on the expression levels of 18 feature genes both in the GSE112676 and GSE112680 datasets. According to it, four of eighteen genes (*BCKDHA, JTB, KYNU, GTF2H5*) have good diagnostic value in the diagnosis of ALS with AUC>0.6 in both the GSE112676 and GSE112680 dataset (**Figures 7A, B**), suggesting that these four genes not only have the prognostic effect but also have the potential diagnostic value.

Branched Chain Keto Acid Dehydrogenase E1 Subunit Alpha (*BCKDHA*), Jumping Translocation Breakpoint (*JTB*), *KYNU*, and General Transcription Factor IIH Subunit 5 (*GTF2H5*) (**Figures 7C, D**) exhibited notable variations in both peripheral blood of ALS patients and the lumbar spinal cord of SOD1^G93A^ mice. Analysis of mRNA expression in SOD1^G93A^ lumbar spinal cord tissue samples revealed an increase in *JTB* and *KYNU* expression, while *BCKDHA* and *GTF2H5* expression were found to be downregulated (**Figure 7E**). Furthermore, to enhance the credibility of these four genes that exhibit differential expression, peripheral blood samples were procured from a cohort of 10 individuals diagnosed with ALS and 10 healthy volunteers to conduct RT-qPCR. The outcomes revealed a significant reduction in mRNA expression levels of *BCKDHA* and *GTF2H5* in the ALS group as compared to the control group (P<0.05), whereas *JTB* and *KYNU* exhibited upregulation (P<0.05) (**Figure 7F**). These findings strongly imply that these four genes possess the potential to function as diagnostic and prognostic biomarkers for ALS.

## 4 Discussion

ALS is a complex pathological process, that involves oxidative stress, mitochondrial dysfunction, excitotoxicity, and neuroinflammatory responses (18). The exact pathogenesis of ALS remains unclear, leading to a lack of practical early diagnostic markers and treatment options, posing challenges for clinical management. Although ALS primarily affects motor neurons in the brain and spinal cord, peripheral blood analysis may provide noninvasive biomarkers. Hence, it is still important to explore new biomarkers and provide new insight.

In our analysis of the GEO dataset, we found two large patient cohorts (GSE112676 and GSE112680) with prognostic information: a microarray dataset from peripheral blood of ALS patients and controls, with a total of, 1117 participants (19). This dataset is the best resource for identifying ALS blood biomarkers. Swindell et al. conducted a meta-analysis and found 752 ALS-increased DEGs with consistent differential expression in both cohorts (GSE112676 and GSE112680) (16). Genes most strongly elevated in ALS blood included ribosomal protein L9 (*RPL9*), ribosomal L24 domain containing 1 (*RSL24D1*), vanin 2 (*VNN2*), mitochondrial amidoxime reducing component 1 (*MARC1*) and kynureninase (*KYNU*).

In this study, genes from the GSE112676 dataset with those involved in the mitophagy process to screen, 4383 DEGs and 22 mitophagy-related DEGs using selection criteria. Subsequently, through single-factor Cox regression analysis of these, 4405 mitophagy-related genes, identified 40 prognosis-related genes. After conducting further LASSO Cox regression analysis, we identified 18 signature genes and used them to establish a risk-scoring model. By stratifying patients into high- and low-risk groups, the relationship between the prognostic model genes and immune infiltration was elucidated. Finally, GSEA and GO/KEGG analyses clarified the pathways enriched by DEGs. ROC curve analysis on the validation set demonstrated the good predictive capability of the model. Finally, we found that four out of the eighteen genes also have prognostic and diagnostic value for ALS. To further determine the expression of these four genes in the disease, we used animal models and clinical samples for the four overlapping genes in the test and training sets. Consistently, *JTB* and *KYNU* were upregulated, while *BCKDHA* and GTF2H5 were downregulated in the ALS group compared to the control group.

Increasing evidence suggests that mitochondrial dysfunction resulting in disrupted energy metabolism is a key pathological feature of ALS (4, 5). The accumulation of impaired mitochondria is regarded as a catalyst for ALS, and concomitantly, deficiencies in mitophagy are also evident in ALS models (20).To date, various genes associated with ALS have been identified to participate in the degradation of damaged mitochondria through the process of mitophagy, such as *p62*, *OPTN*, and *TBK1* as discussed previously (21, 22). A study showed that mutant SOD1 affects mitophagy by preventing autophagy receptors from binding to damaged mitochondria (23). Wang et al. found that *C9ORF72* helps regulate energy balance by stabilizing mitochondrial complex I (24). Mutations in *TARDBP* lead to TDP-43 buildup in mitochondria. A recent study by Yu et al. suggests a link between TDP-43, mtDNA, and inflammation in ALS development (25). The VCP protein is crucial for maintaining mitochondrial quality control and mutant VCP disrupts the labeling of mitochondria for mitophagy (26, 27). These discoveries suggest that impaired mitochondrial transport may play a role in the development of ALS. However, investigations into mitophagy in ALS have been limited to morphological examinations of autophagosomes and mitochondrial changes. The connection between mitophagy and ALS is not fully understood, and mitophagy-related genes in ALS have not been thoroughly studied using bioinformatics analysis. This study aims to create a prediction model related to mitophagy and explore the relationship between mitophagy-related genes and immune infiltration in ALS to uncover potential immune mechanisms and identify new biomarkers.

*JTB* regulates cell proliferation during mitosis and can inhibit TGFB1-induced apoptosis (28). Moreover, *JTB* affects cell proliferation and growth by increasing AURKB activity (29), and its overexpression induces mitochondrial swelling and reduces mitochondrial membrane potential. The results revealed increased mRNA levels of *JTB* in the serum of ALS patients and the lumbar spinal cord of SOD1^G93A^ mouse models, consistent with the bioinformatics analysis. Other studies have also reported mitochondrial defects and impairment of the autophagy pathway in ALS patients (23, 30). Furthermore, research has shown that overexpression of JTB in cells leads to perinuclear aggregation and swelling of mitochondria, accompanied by a significant decrease in membrane potential, as detected by JC-1 staining (28). Thus, the overexpression of *JTB* in ALS may induce mitochondrial damage, suggesting a novel key factor in mitochondrial dysfunction associated with ALS.

*BCKDHA*, together with *BCKDHB*, forms the E1 subunit of the mitochondrial branched-chain alpha-keto acid dehydrogenase (BCKD) complex (31). This study showed decreased mRNA levels of *BCKDHA* in the serum of ALS patients and the lumbar spinal cord tissue of SOD1^G93A^ mouse models, consistent with the bioinformatics analysis. *BCKDHA* and *VDAC1* are both mitochondrial proteins that jointly participate in lipid metabolism processes. Research has indicated that *BCKDHA* and *VDAC1* can undergo immunoprecipitation with APOE in mouse liver extracts (32). This interaction plays a crucial role in the energy production processes of mitochondria and helps the liver to adapt to its energy demands. Furthermore, additional studies suggest that *BCKDHA* (33) induces β-cell mitochondrial dysfunction, stress signal transduction, and cell apoptosis related to type 2 diabetes mellitus (T2DM). Since there are a limited number of studies on *BCKDHA* and ALS, our study may provide some insights for future research.

*KYNU* is an enzyme involved in the kynurenine pathway (KP), which generates metabolites with immunomodulatory properties. The activation of the KP and the subsequent overproduction of the KP metabolite quinolinic acid due to neuroinflammation are prevalent characteristics in various neurodegenerative disorders, such as ALS. Mutations in the *WARS* and *KYNU* genes negatively impact protein synthesis and cell viability, and cause neurite degeneration in neuronal cells and rat motor neurons (34). The experimental results indicate elevated mRNA levels of *KYNU* in serum from ALS patients and lumbar spinal cord tissue of SOD1^G93A^ mouse models compared to healthy individuals. This result ties well with previous studies wherein the presence of a functional KP in NSC-34 cells, with KYNU and *TDO2* being among the components of the KP in these cells (35). Jennifer et al. identified five genes within the KP (*AFMID, CCBL1, GOT2, KYNU, HAAO*) that exhibit either unique protein-altering variants or an accumulation of rare protein-altering variants in sporadic ALS cases compared to controls (34). Swindell found *KYNU* genes most strongly elevated in ALS blood (16). Our results were consistent with these findings and we also showed that a higher expression of *KYNU* is associated with a better prognosis. Further studies are still needed to clarify why the expression patterns of those genes are different even though they trigger the same canonical pathway of pyroptosis.

*GTF2H5* encodes a subunit of the transcription/repair factor TFIIH, which plays a role in gene transcription (36). *GTF2H5* and *PINK1* are involved in gene expression, transcription pathways, and RNA polymerase II transcription. The results indicate decreased mRNA levels of *GTF2H5* in serum from ALS patients and lumbar spinal cord tissue of SOD1^G93A^ mouse models compared to healthy individuals. PHB2 depletion disrupts the stability of PINK1 in mitochondria, thereby blocking the recruitment of PRKN/Parkin, ubiquitin, and OPTN to mitochondria, resulting in inhibited mitophagy (37). Furthermore, *GTF2H5* deficiency leads to resistance to free celastrol, a compound derived from Tripterygium (TP) (38). Therefore, decreased expression levels of *GTF2H5* in ALS patients may lead to impaired mitophagy.

Neuroinflammation serves as one of the pathological hallmarks of ALS. After central nervous system (CNS) injury, various types of innate and adaptive immune cells from the peripheral circulation, including granulocytes, monocyte-derived macrophages, lymphocytes, and natural killer (NK) cells, can be recruited across the blood-brain barrier (BBB) (39, 40). Post-mortem analysis of ALS patient tissue revealed infiltration of peripheral cells, such as CD4+ and CD8+ T cells, macrophages, and NK cells, into multiple CNS regions, indicating the involvement of immune-mediated events in ALS pathogenesis (41, 42). An annotation-based enrichment analysis revealed that DEGs associated with neutrophils were increased in patients with ALS (16).Our study identified peripheral blood immune cells potentially associated with ALS prognosis, including B naive cells, CD4 naive T cells, CD8 T cells, M0 and M2 macrophages, and neutrophils. Shi et al. also used the GSE112676 and GSE112680 datasets to construct a risk model involving four genes (*TRPM2, ROCK1, HSP90AA1, and HSPA4*) *(* 43). Moreover, external validation from dataset GSE112681 confirmed the predictive power of the model. *TRPM2* down-regulation and *ROCK1* up-regulation were also found at the initial stage of our study, but they were not aggregated into further studies due to different directions of subsequent research. Currently, the establishment of prediction models for ALS solely considers DEGs as a single factor, disregarding the variability of the disease. By constructing a gene model, this study not only improves the accuracy of feature gene selection but also enhances the specificity of gene screening.

Currently, there is limited research on mitophagy and immune infiltration in ALS. The expression levels of four key genes in peripheral blood and lumbar spinal cord tissues of ALS mic and found statistically significant expression differences, indicating that *JTB, KYNU, BCKDHA*, and *GTF2H5* in peripheral blood can serve as practical clinical biomarkers for diagnosing ALS patients. However, this study also has some limitations (1): Even though the prognostic risk score performed well, large prospective cohort studies are still needed to validate it; (2) As mitophagy-related genes continue to be discovered, the model needs constant improvement; (3) The expression levels of four prognostic model genes were verified using qRT-PCR *in vitro* through the collection of clinical samples and mouse models, without delving into the underlying mechanism. Hence, additional research is warranted to elucidate the molecular mechanisms involved.

In conclusion, we have created and verified a new prognostic predictive risk score for ALS based on four mitophagy-related genes. This risk score demonstrated its independence as a prognostic factor for ALS outcomes. Furthermore, the analysis of the correlation between these four genes and immune infiltration in ALS indicated a potential involvement of the interaction between mitophagy-related genes and immune cell infiltration in the regulation of ALS pathogenesis. These results offer a fresh perspective on the roles of mitophagy and immune infiltration in ALS and lay the groundwork for further investigations.

## Data availability statement

The original contributions presented in the study are included in the article/**Supplementary Material**. Further inquiries can be directed to the corresponding author.

## Ethics statement

The studies involving humans were approved by The Ethics Committee of the Chinese People’s Liberation Army (PLA) General Hospital. The studies were conducted in accordance with the local legislation and institutional requirements. The participants provided their written informed consent to participate in this study. The animal study was approved by The Institutional Animal Care and Use Committee of the Chinese PLA General Hospital. The study was conducted in accordance with the local legislation and institutional requirements.

## Author contributions

RD: Conceptualization, Data curation, Investigation, Writing – original draft, Writing – review & editing. PC: Formal analysis, Methodology, Software, Visualization, Writing – review & editing. ML: Data curation, Formal analysis, Software, Writing – review & editing. YZ: Data curation, Software, Writing – review & editing. ZH: Data curation, Investigation, Methodology, Writing – review & editing. XH: Conceptualization, Funding acquisition, Supervision, Validation, Writing – review & editing.
