# Supplementary material for: Developing a novel immune infiltration-associated mitophagy prediction model for amyotrophic lateral sclerosis using bioinformatics strategies
Source: Front Immunol. 2024 Mar 27;15:1360527. doi: 10.3389/fimmu.2024.1360527 (PMC11005030; doi:10.3389/fimmu.2024.1360527)
Supplement: Supplementary file 1 [file Image_1.pdf]

Supplementary Material

Article Title

Developing a novel immune infiltration-associated mitophagy prediction model for amyotrophic lateral sclerosis using bioinformatics strategies

Rongrong Du[1,2], Peng Chen[3,4], Mao Li[2], Yahui Zhu[2,3], Zhengqing He[5], Xusheng Huang [1,2,3*]

[1] School of Medicine, Nankai University, Tianjin, China.

[2] Department of Neurology, The First Medical Center, Chinese PLA General Hospital, Beijing, China.

[3] Medical School of Chinese PLA, Beijing, China.

[4] Department of General Surgery & Institute of General Surgery, The First Medical Center of Chinese PLA General Hospital, Beijing, China.

[5] Department of Neurology, Beijing Friendship Hospital, Capital Medical University, Beijing, China.

*** Correspondence:**
Xusheng Huang*

lewish301@163.com

Supplementary Figure

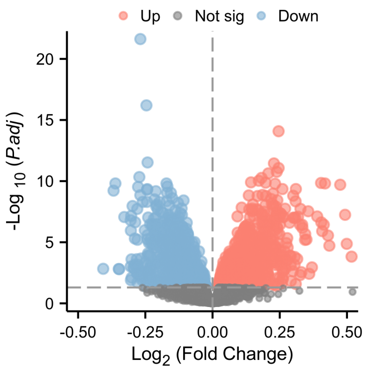

Supplementary Figure 1. The differentially expressed genes in the validation dataset.
