# Supplementary material for: Developing a novel immune infiltration-associated mitophagy prediction model for amyotrophic lateral sclerosis using bioinformatics strategies
Source: Front Immunol. 2024 Mar 27;15:1360527. doi: 10.3389/fimmu.2024.1360527 (PMC11005030; doi:10.3389/fimmu.2024.1360527)
Supplement: Supplementary file 7 [file Table_6.docx]

Supplementary Material

# Supplementary Tables

Supplementary Table 1. The sequences of primers.

| **Gene symbol** | **Primer** | **Sequences (5’-3’)** |
| --- | --- | --- |
| JTB | Forward primer | GCGCTTTCACCTTAAAACTCTG |
|  | Reverse primer | TGGAAGTTAGAACACGGAGTACA |
| BCKDHA | Forward primer | CTCCTGTTGGGACGATCTGG |
|  | Reverse primer | CATTGGGCTGGATGAACTCAA |
| KYNU | Forward primer | GTCAAGCCTGCGTTAGTGG |
|  | Reverse primer | GGAGGGTTTGAAATTCGGAATCC |
| GTF2H5 | Forward primer | ATGGTCAACGTGCTGAAAAGG |
|  | Reverse primer | AAGACGTGCGTGTCATCAATG |
| GAPDH | Forward primer | AGGTCGGTGTGAACGGATTTG |
|  | Reverse primer | TGTAGACCATGTAGTTGAGGTCA |
